# The Impact of Minimal Residual Disease Measurement in the Management of Chronic Lymphocytic Leukemia

**DOI:** 10.3390/cancers17101708

**Published:** 2025-05-20

**Authors:** Megan R. Greenberg, Thomas Lucido, Kritika Singh, Joanna M. Rhodes

**Affiliations:** 1 Department of Medicine, Rutgers Robert Wood Johnson Medical School, New Brunswick, NJ 08901, USA; mg1892@rwjms.rutgers.edu (M.R.G.); tl841@rwjms.rutgers.edu (T.L.); krs228@rwjms.rutgers.edu (K.S.); 2 Rutgers Cancer Institute, New Brunswick, NJ 08901, USA

**Keywords:** chronic lymphocytic leukemia, measurable residual disease, minimal residual disease, UMRD

## Abstract

*Background:* The treatment of chronic lymphocytic leukemia (CLL) has advanced considerably in recent years. Bruton’s tyrosine kinase inhibitors (BTKis) and B-cell lymphoma 2 inhibitors (BCL2is) such as venetoclax have largely supplanted chemoimmunotherapy for both frontline and relapsed CLL. With the introduction of additional innovative agents and regimens, the clinical role of measurable residual disease (MRD) has become complicated. *Methods:* In this article, we will review the existing literature on MRD and its utility in the management of CLL. We will review the definitions of MRD, review MRD detection methods, and discuss the use of MRD in the current CLL treatment landscape. In doing so, we will clarify the present and conceivable future roles of MRD for the treatment of CLL. *Conclusions:* MRD is a powerful tool to assess response to CLL therapies, and can be prognostic with certain treatment regimens, such as fixed-duration venetoclax-based treatment. While we do not recommend MRD testing in routine clinical practice, we believe it has an important role in assessing treatment response and will be utilized routinely in the future. Further studies to incorporate MRD into treatment strategies for CLL are ongoing and will help to inform how we utilize it in clinical practice.

## Simple Summary

The management of chronic lymphocytic leukemia (CLL) continues to advance with the advent of new pharmacologic agents. As the treatment paradigm shifts, so does how we use the technologies available to help make decisions. For example, the utilization of technologies that allow us to detect the presence of disease after a treatment has been given, termed measurable residual disease (MRD), has been demonstrated to be an important endpoint in clinical trials for CLL treatment, but has not been introduced in routine clinical practice. In this review, we discuss methods for MRD measurement and review the evidence for MRD use in the treatment of CLL. In doing so, we aim to highlight the role of MRD in the current landscape of CLL treatment.

## 1. Introduction

Chronic lymphocytic leukemia (CLL) is a low-grade B-cell lymphoproliferative disorder and the most prevalent adult leukemia in Western countries. It was estimated that in the United States in 2024, 20,700 people would be diagnosed with CLL and 4440 individuals would die of the disease [1]. There are multiple ways to risk stratify patients, including the Rai staging system, the Binet staging system, and the CLL international prognostic index [2,3]. Additionally, there are prognostic biomarkers associated with the disease, including an unmutated immunoglobulin heavy-chain variable gene (IGHV), as well as TP53 aberrations (TP53 mutations or Del (17p)) [4]. Treatment for CLL is initiated based on recommendations from the Internal Workshop on CLL (iwCLL) criteria, including the presence of B symptoms, hepatosplenomegaly, massive lymphadenopathy, or cytopenias. Response assessment also utilizes these clinical parameters. However, patients who have achieved a complete clinical response can still have detectable measurable residual disease (MRD) [2].

MRD is generally considered a highly sensitive marker of disease burden in CLL. The use of MRD in clinical decision-making for patients with CLL is a continuously evolving subject. It is known that MRD measurement has shown excellent prognostic value in patients treated with chemoimmunotherapy (CIT) [5]. However, the treatment landscape is rapidly changing in CLL, and the standard of care has largely transitioned towards targeted agents and cellular therapies. The significance of MRD status in these settings continues to evolve.

The most recent guidelines established by the iwCLL 2018, as well as the European Society of Medical Ongology (ESMO) consensus conference in 2016, recommend the use of MRD in clinical trials, but not yet in routine clinical practice [2,6]. The National Comprehensive Cancer Network (NCCN) clinical practice guidelines updated in a 2025 note that MRD assessment is not recommended outside of clinical trials for response evaluation at this time [4]. Despite its lack of use in clinical practice, MRD remains an important tool in our armamentarium, and further research will determine its utility in clinical practice. In this review, we set out to define MRD, review methods for detection, summarize current evidence for the use of MRD in clinical trials, and discuss how recent updates may affect the developing role of MRD testing in routine clinical practice.

## 2. Definition and Detection of MRD

A 174-member expert consensus panel published recommendations regarding the definition and use of measurable residual disease (MRD). Although the terms “minimal residual disease” and “measurable residual disease” are often used interchangeably, the panel recommends “measurable residual disease” as the standard, given its more objective terminology. Similarly, the panel recommends the use of “undetectable MRD” (uMRD) to describe the inability to detect measurable disease below a specific reporting threshold, as it is less ambiguous than “MRD negative” [7]. The nomenclature for MRD thresholds is defined by the upper limit of the disease. For instance, MRD4 represents a threshold of 10^−4^ leukocytes (less than 1 CLL cell in 10,000 or 0.01%), while MRD5 represents a threshold of 10^−5^ leukocytes (less than 1 CLL cell in 100,000 or 0.001%), and so on [7]. According to the iwCLL guidelines, MRD4 is an acceptable threshold to define UMRD response [2].

It is important to report the compartment tested for MRD, since there is the possibility of discordant MRD results between the peripheral blood (PB) and the bone marrow (BM) [2]. Generally, if the PB is found to have residual CLL, the BM does not need to be tested. However, if uMRD is reported in the PB, it may be prudent to confirm the results with testing of the BM, given that some treatment regimens preferentially clear disease in the peripheral blood [2]. For instance, studies of monoclonal antibody-based regimens have shown a discordance between results reported in the PB in comparison with those in the BM [7].

It is also crucial to account for the method of assessment of MRD. MRD can be evaluated with flow cytometry (FC), allele-specific oligonucleotide real-time quantitative polymerase chain reaction (ASO-RQ-PCR), next-generation sequencing (NGS), and circulating tumor DNA (ctDNA) via droplet digital PCR (ddPCR) (Table 1). All these testing modalities have different sensitivities and availability in practice [7,8].

FC is a highly standardized and widely used approach in clinical trials to assess MRD, and can be performed in routine clinical practice [9]. It involves measuring the florescence of a large, fresh sample and analyzing cells via various markers. A common FC-based assay comprises a core panel of six markers (i.e., CD19, CD20, CD5, CD43, CD79b, and CD81) [2,10]. While a four-color assay has been the historical gold standard, different assays exist, each with unique advantages and disadvantages [7,10]. One single-tube assay, which has been identified and validated by the European Research Initiative on CLL (ERIC), can reliably quantitate CLL cells to MRD5, and can be adapted to most laboratories using cytometers with six or more colors [10]. Most new flow cytometry instruments offer eight- or ten-color analysis, allowing for the simultaneous analysis of other markers [10].

ASO-RQ-PCR is a useful technology for identifying leukemia-specific rearrangements. It takes patient-specific primers to detect the junctional region of rearranged immunoglobulin-heavy (IgH) and light-chain genes and T-cell receptor (TR) genes. The detection limit varies by patient and must be determined before the start of treatment. It is well standardized, with the ability to detect up to MRD5 [11,12]. Unlike FC, it does not require a fresh sample. However, it requires patient specific primers, is labor intensive, and expensive. As a result, it is used in clinical trials but is not used frequently in clinical practice [13].

NGS-based assays such as the clonoSEQ assay can also be used to detect MRD and have been incorporated into clinical trial design [10,14]. The process involves fragmenting DNA/RNA, adding adapters, sequencing the libraries, and reassembling them to form a genomic sequence. The clonoSEQ assay identifies rearranged IgH (VDJ), IgH (DJ), IgK, and IgL receptor gene sequences, as well as translocated BCL1/IgH (J) and BCL2/IgH (J) sequences [15,16]. NGS is a validated approach, with a sensitivity reaching MRD6, and has shown concordance with flow cytometry-based assays [10,14,15]. Advantageously, this method also does not require patient-specific primers, making it more universally applicable to routine clinical practice than ASO-RQ-PCR [14]. It does require a pretreatment sample. The improved sensitivity of NGS may translate to improved prognostic discrimination, but more studies are needed to determine what level and test are optimal [5,17].

ctDNA-based MRD is another emerging approach, which can be analyzed with ddPCR [9,18]. ddPCR is a method in which the target can be directly quantified (via counting the rate of droplets containing the target gene) without a reference sample [19]. It is accurate and precise, especially when the target concentration is very low, making it theoretically ideal for the purposes of MRD quantification [19]. It has been shown to detect at least to the level of MRD5, and it has the potential to be performed at low costs with a fast turnaround time, making it an attractive modality in clinical practice [7,9]. While ctDNA approaches have, thus far, shown high concordance with flow cytometry, and the optimal approach may include the utilization of both methods in conjunction, more validation studies are needed [9,18,20].

## 3. MRD with Continuous Treatment with Targeted Agents

The approval of targeted agents has changed the standard of care for CLL [21]. Trials have demonstrated improvements in progression-free survival (PFS) and overall survival (OS) with the use of targeted agents when compared to CIT, leading to the broad uptake of their use when availabe (Table 2) [22,23,24]. Many of these agents are to be taken continuously until unacceptable toxicity or disease progression. There are limited data on uMRD rates with continued single-agent BTK inhibitor therapy. A phase II CLL study of elderly patients or patients with TP53 mutations receiving ibrutinib monotherapy showed low rates of uMRD, but impressive five-year PFS rates [25]. Similarly, low rates of UMRD were seen with the use of the pivotal drug E1912 (ibrutinib-rituximab) vs. FCR [23,26] and ELEVATE TN (acalabrutinib, acalabrutinib-obinutuzumab, chlorambucil-obinutuzumab) [27,28], despite improvements in PFS. Therefore, the use of MRD in the continuous targeted treatment setting is not advised.

## 4. MRD in Frontline Fixed-Duration Treatment of CLL with Targeted Treatments

The use and understanding of MRD is of particular importance for time-limited treatment of CLL. Using a time-limited treatment approach may confer deep responses without the continued risk of long-term toxicities. Additionally, the loss of continuous selective pressures may prevent the development of resistance mutations. Several trials have started to explore the use of UMRD as an endpoint to help determine the optimal duration of therapy. Notable studies assessing time-limited regimens include CLL14, FLAIR, CAPTIVATE, GLOW, CLL-13, BOVEN, and AVO, which are outlined below.

*CLL14*

CLL14 was a pivotal randomized phase 3 trial leading to the approval of venetoclax and obinutuzumab (VO) for the treatment of CLL in a first-line setting. Patients with previously untreated CLL and coexisting conditions were randomized to time-limited treatment with 12 cycles of VO or chlorambucil-obinutuzumab (CO). The primary outcome was PFS. uMRD rates (assessed using MRD4) were included in the secondary endpoints, via ASO-PCR in the PB and BM [29]. Exploratory endpoints included uMRD rates at a cutoff of MRD4, MRD5, and MRD6 via NGS. At four years, the PFS rate was 74% in the VO arm and 35.4% in the CO arm, and the PFS benefit was still observed in patients with high-risk features (TP53 mutations and IGHV status). Three months after treatment completion, 40% of patients in the VO arm had UMRD at a threshold of MRD6 via NGS, compared to 7% in the CO arm. Reappearance of detectable MRD (dMRD) after VO was significantly slower than after CO [30]. Furthermore, at six years, the PFS rate was 53% in the VO arm, reinforcing the sustained benefit of the regimen. At five years, 7.9% of those in the VO arm had sustained UMRD, compared to 1.9% in the CO arm. The end-of-treatment MRD status was associated with PFS and OS. Interestingly, in the VO arm, 6-year PFS rates improved with the achievement of lower MRD thresholds at the end of treatment. A similar association between end-of-treatment MRD status and PFS was observed in patients, regardless of IGHV status or TP53 mutations [31].

*FLAIR Trial*

The FLAIR trial compared ibrutinib and venetoclax (IV) to fludarabine, cyclophosphamide, and rituximab (FCR) in fit patients between 18 and 75 years old with CLL in the frontline setting [32]. The primary outcome of the study was PFS. MRD4 was used as a threshold for uMRD and was measured via flow cytometry. Patients received ibrutinib for 2 months, followed by the combination of ibrutinib and venetoclax. The duration of IV therapy was double the time it took until a UMRD stopping algorithm was complete, there was toxicity, or there was disease progression. In the stopping algorithm, starting at 1 year, patients had their MRD measured via the PB every six months, and treatment was continued for double the duration of the time from randomization to the time to first UMRD in the PB, or for up to 6 years of therapy. With a median follow-up of 43.7 months, the estimated 3-year PFS was 97.2% for patients treated with IV, compared to 76.8% for FCR. PFS was worse for patients with unmutated IGHV treated with FCR compared to IV. Three-year OS rates were 98% for patients treated with IV, compared to 93% for those treated with FCR. The uMRD rate in the bone marrow was 41.5% for IV and 48.3% for FCR at 9 months post randomization and was 61.9% for IV at any time. Most patients received 2–3 years of treatment with IV [32]. These data demonstrate the potential importance of uMRD as a method to either minimize the treatment duration for fast responders or maximize the depth of response for patients potentially needing longer durations of therapy, allowing for personalization of therapy to potentially maximize response.

*GAIA/CLL13*

CLL13 is an ongoing multicenter phase 3 clinical trial that is evaluating the efficacy and safety of venetoclax combinations vs. chemoimmunotherapy (FCR or bendamustine-rituximab (BR)) as a frontline treatment for fit patients with CLL without *TP53* mutations/deletions and del(17p). Patients were randomized in a 1:1:1:1 fashion to receive a time-limited course of CIT, venetoclax-rituximab (VR), VO, and VO + ibrutinib (VOI). The primary endpoints of the study were uMRD in the PB at month 15 and PFS at four years. MRD was measured at months 9, 12, and 15 via FC of the PB, and uMRD was defined as MRD4. For patients in the VOI group, ibrutinib was stopped when uMRD was achieved on two consecutive measurements. The rate of uMRD at 15 months was significantly higher in patients receiving VOI (92%) and VO (86.5%), compared with those receiving CIT (52%). The 4-year PFS rates were significantly longer in the VOI (85.5%) and VO (81.8%) groups compared to the VR (70.1%) and CIT (62.0%) groups. For patients with mIGHV, there was no significant difference in PFS for patients treated with VOI vs. VO. In a subgroup analysis, VOI yielded longer PFS than the VO subgroup when compared directly; however, the rates of infection and adverse cardiac events were higher in the VOI group [33].

*CAPTIVATE*

The CAPTIVATE trial is a phase 2 trial evaluating first-line treatment with IV in fit patients aged 18–70. It consists of an MRD cohort and a fixed-duration (FD) cohort. In the MRD cohort, uMRD-confirmed patients were randomized to receive either placebo or ibrutinib until confirmed MRD relapse, and the unconfirmed UMRD patients were randomized to receive ibrutinib or IV until disease progression or unacceptable toxicity. UMRD was confirmed using a threshold of MRD4 via flow cytometry, requiring two serial assessments in both PB and BM [34].

In the MRD-based cohort, after 12 cycles of IV, the best uMRD response rates were 75% (PB) and 68% (BM). In the confirmed uMRD population, there was no significant difference in one-year DFS between the placebo and ibrutinib. The 30-month PFS rates in the confirmed U-MRD population were 95% with the placebo and 100% with ibrutinib. The 30-month PFS rates in the not-confirmed uMRD population were 95% with ibrutinib monotherapy and 97% with IV [34].

In the FD cohort, the best UMRD rates were 77% (PB) and 60% (BM). The 24-month PFS rates were high in the all-treated population (97–100%), regardless of clinical response and whether uMRD was achieved [35]. A pooled analysis of patients treated with FD IV showed that uMRD rates were slightly higher in patients with one or more high-risk features (88% in PB and 72% in BM) than in those without high-risk features (70% in PB and 61% in BM). This may be largely accounted for by patients with unmutated IGHV status, given that they are the bulk of the high-risk subgroup and demonstrated similar results, in contrast to those with TP53 mutations, in whom the rate of uMRD was much lower than in those without high-risk features. The 36-month OS rates in this analysis were >95%, regardless of high-risk features [36].

*GLOW*

The GLOW trial evaluated patients in a 1:1 fashion to receive fixed-duration IV vs. CO in the frontline setting for non-del (17p) CLL. It enrolled patients over the age of 65, or patients aged 18–64 with a Cumulative Illness Rating Scale score greater than 6 [37,38]. MRD was assessed via NGS/ClonoSEQ, and separately by eight-color flow cytometry in the PB and BM, with MRD4 utilized as the UMRD threshold. The 42-month PFS rates were improved in patients treated with IV compared with those treated with CO (74.6% vs. 24.8% for CO). For patients treated with IV and mutated IGHV, regardless of their MRD status at the end of treatment, the PFS rate at 2 years was more than 90%. In contrast, patients treated with IV and unmutated IGHV who achieved UMRD at the end of treatment had a PFS rate of over 90%, while patients who had not achieved UMRD showed a 67% PFS rate [39]. A recent analysis performed by the investigators demonstrated that IV treatment led to higher MRD5 rates in the PB and BM (43.4%, 40.6%) compared to CO treatment (18.1%, 7.6%), and that these uMRD responses were more durable for patients treated with IV. Despite these differences, there was no significant difference in PFS for patients treated with IV, regardless of EOT +3 uMRD status in the BM (PFS EOT +12: MRD4 96.3% vs. 93.3%. Previously, uMRD has been prognostic for improvements in PFS and OS in other fixed-duration venetoclax-based regimens, and this finding will need further investigation [38]. This highlights that MRD may have differing significance based on the regimen and may need to be utilized differently depending on treatment. It is notable that the dMRD group contained more patients with favorable risk factors, such as mIGHV, which suggests that disease biology may also play a role in the significance of uMRD in influencing long-term outcomes.

*Triplet Combinations:*
*AVO:*

In a phase 2 study of patients with treatment-naive CLL, the utility of a time-limited treatment with acalabrutinib, venetoclax, and obinutuzumab (AVO) is being studied. Treatment-naïve CLL patients with any genetic risk profile are included. Those with *TP53*-aberrant CLL are being analyzed in a separate cohort. The primary endpoint of the study is BM uMRD, as defined by MRD6 via clonoSEQ. If uMRD is achieved at C16, patients are to discontinue therapy. Those not achieving uMRD are to continue treatment through C24 for re-evaluation. Thus far, 68 pts have been evaluated. For those with unmutated *TP53*, 86% achieved uMRD at C16 and subsequently stopped treatment. For those with *TP53*-aberrant CLL, 83% achieved BM-uMRD at C16. Combined, 93% of patients experienced PFS for a duration of analysis of almost three years. Currently, there is no consensus on the best initial therapy for patients with del17p/TP53, and it is often debated whether time-limited therapy is appropriate in this population. This study indicates that AVO may be a effective, time-limited treatment for higher-risk patients, and MRD could potentially guide response to therapy for these patients [40].

*BOVen*

One key question is how MRD can be used to tailor treatment duration. In a phase 2 trial multicenter BOVEN study, MRD was used to guide treatment duration of the combination of zanubrutinib, venetoclax and obinutuzumab for treatment-naïve CLL patients. Patients received zanubrutinib and obinutuzumab for two cycles and started venetoclax during cycle three. Patients continued to the triplet combination through 8–24 cycles, and stopped treatment if UMRD4 was detected in the PB and BM. At a median follow-up of 25.8 months, 89% of patients had uMRD responses in PB and BM and stopped therapy after a median of 10 cycles. After 15.8 months of follow-up, 94% of patients had uMRD demonstrating that uMRD directed treatment strategies are feasible [41]. Further long-term follow-up is needed to determine the duration of these responses, as well as the time to next treatment, to demonstrate the efficacy of this strategy. Interestingly, the BOVen trial also included a post hoc analysis which found that an MRD reduction to 1/400th of the baseline (ΔMRD400) could predict uMRD at the end of cycle eight. It identified patients with delayed bone marrow MRD clearance despite a longer treatment duration, thus suggesting that this can serve as a high-risk feature independent of traditional high-risk genomic markers. ΔMRD400 could conceivably serve as another tool to guide the duration of therapy in the future, with the potential to further limit therapy duration in fast responders [41].

*AMPLIFY*

Recently, the AMPLIFY phase 3 trial reported the results of the fixed-duration regimen of venetoclax and a covalent BTKi for first-line CLL treatment. The study included fit patients without del (17p) or TP53 mutations [42]. Patients were randomized in a 1:1:1 fashion to receive a fixed-duration treatment of acalabrutinib and venetoclax, fixed-duration AVO, or CIT with the investigator’s choice of FCR or BR. The primary outcome of the study was PFS. uMRD in the PB, defined as MRD4, was a secondary outcome of the study. uMRD was assessed in the PB with FC at the start of cycle 9 in AV, the start of cycle 10 of AVO, and at 12 weeks after the start of cycle 6 for CIT. Patients treated with AV had a PFS of 76.5% at 3 years, in comparison to 83.1% with AVO and 66.5% with CIT. AV additionally had an overall survival benefit in comparison to CIT. uMRD was achieved in 25.8%, 66.4%, and 51% of patients treated with AV, AVO, and CIT, respectively.

UMRD was achieved in a modest proportion of patients treated with AV. The addition of obinutuzumab to treatment bolstered the proportion of patients achieving uMRD in this study, but this treatment comes with the cost of higher rates of infections and other adverse events. That said, the data suggest that, despite an improvement in uMRD, there is only a minimal potential PFS advantage of AVO in comparison to AV. Indirect comparisons should be taken with caution, but they highlight the differences in IMRD rates for AV in AMPLIFY in comparison to what was seen for IV in CAPTIVATE. Longer follow-up will be needed to further evaluate the impact that uMRD has on this regimen’s long-term efficacy.

## 5. MRD for Relapsed/Refractory Disease with Targeted Agents

In patients with relapsed/refractory CLL requiring treatment, second-line therapy is usually selected based on the class of first-line therapy, duration of remission, reason for discontinuation, and presence of acquired resistance mutations [4]. The utility of MRD as a prognostic marker is also being explored as an endpoint in patients receiving targeted agents for the study of relapsed/refractory CLL, for example, in the MURANO and CLARITY trials.

*MURANO*

The MURANO trial is a pivotal phase 3 clinical trial comparing two years of venetoclax-rituximab (VR) treatment to six months of bendamustine and rituximab (BR) treatment for patients with CLL in the relapsed/refractory setting. The primary outcome was PFS and the rate of MRD was included as a secondary outcome using a threshold of MRD4. MRD was assessed in the PB and BM with ASO-RQ-PCR and FC [43]. At a follow-up of 5 years, the survival benefits of VR compared to BR were sustained, with a median PFS of 53.6 months with VR compared to 17 months with BR. Furthermore, the MRD doubling time with VR was 93 days, compared to 53 days with BR. The MRD status at end of treatment with VR was a strong predictor of PFS, as well as OS. The 3-year PFS was 61.3% in those achieving uMRD at the end of treatment, compared to 40.7% in the low-MRD-positive patients; nearly all patients with a high-MRD-positive status had disease progression prior to two years from the EOT. In the VR arm, while patients with unmutated IGHV or TP53 mutations were able to achieve high rates of uMRD, they showed a faster MRD doubling time after treatment completion [44]. In the final 7-year follow-up, similar trends were shown. The 7-year PFS rates were 23% with VR, compared to 0% with BR. VR-treated patients without progressive disease who achieved uMRD at the end of treatment had a median PFS of 52.5 months, compared to 18 months in patients who were MRD-positive [45]. Thus, uMRD status can be prognostic in this setting, but disease biology continues to play a role in the depth and duration of uMRD responses.

*CLARITY*

CLARITY was a phase 2 trial evaluating IV in a relapsed/refractory setting, with a primary endpoint of achieving MRD4 via flow cytometry in both the PB and BM after 12 months of combination therapy. The duration of treatment was based on the MRD response to therapy. The duration was 14 months for patients with uMRD in both the PB and BM at 8 months, and the duration was 26 months for patients achieving uMRD at 14 months or 26 months. For patients who still had detectable MRD at month 26, venetoclax was discontinued, and ibrutinib was continued until progression. After 12 months of IV, UMRD was achieved in 36% of patients in the BM and 53% of patients in the PB. Out of all patients, 89% responded, and 51% achieved complete remission. A total of 44% of the patients achieved uMRD at month 26, demonstrating continuous improvement in the depth of MRD reduction with a longer duration of treatment for some patients [46]. In an exploratory analysis, the achievement of UMRD4 after 6 months or a 2-log reduction in MRD levels after 2 months of treatment with IV resulted in sustained MRD and clinical response at 3 years [47], highlighting the role of MRD in venetoclax-based treatment.

*VENICE*

VENICE-1 was a phase 3 trial that evaluated venetoclax monotherapy in the relapsed/refractory setting. This international, multicenter trial was a single-arm design and excluded patients with del (17p) and TP53-abberations. Patients received daily venetoclax monotherapy for up to 108 weeks and were subsequently followed up for 2 years after discontinuation. Patients were stratified by prior treatment with BCRi therapy. MRD in the PB via NGS was assessed in all patients at baseline, week 24, and week 48, and UMRD was defined as MRD4. The primary outcome of the study was the complete remission rate, defined by iwCLL guidelines. Thirty-five percent of BCRi-naïve patients experienced complete remission, compared to 29% of BCRi-pretreated patients. UMRD was experienced at similar rates in both the BK-naïve and treated groups [48].

## 6. MRD with Cellular Therapy

Allogeneic stem cell transplantation is a possibility for fit patients in the treatment of CLL, but given the efficacy of targeted therapies, it is generally considered only after prior lines of therapy with BTK inhibitor- and venetoclax-based regimens [4]. Data regarding UMRD and transplantation are limited. The use of chimeric antigen receptor (CAR) T-cells for relapsed/refractory disease is also under continued development in clinical trials, along with the assessment of MRD [49].

*CLL3X*

CLL3X was a German CLL phase 2 trial investigating the outcomes of reduced-intensity conditioning (fludarabine/cyclophosphamide-based) allogeneic stem cell transplant in patients with poor-risk CLL. MRD was assessed via flow or ASO-RQ-PCR, and uMRD was defined as MRD4. For 52 out of 100 eligible patients, MRD monitoring was available, and 27 (52%) of the MRD-monitored patients were alive and achieved uMRD one year after transplant. The UMRD status at one year was found to be a prognostic factor for long-term clinical remission [50]. Follow-up at six years showed a 58% OS and 38% event-free survival, independent of the presence of poor-risk mutations [51].

*TRANSCEND CLL004*

The TRANSCEND CLL004 trial was a phase 1–2 study evaluating the safety and efficacy of lisocabtagene maraleucel (liso-cel), a CD19-directed CART cell product. It showed that liso-cel induced complete response or remission in patients with relapsed or refractory CLL or small lymphocytic leukemia, with a manageable safety profile. A threshold of MRD4 was used, assessed via clonoSEQ. The uMRD rate was 63% in the PB and 59% in the BM. UMRD was associated with a longer PFS compared to detectable MRD, and all patients who reached CR or PR reached UMRD in both the blood and marrow [49]. Liso-cel has recently undergone accelerated approval by the FDA for the treatment of CLL in patients who have received at least two prior lines of therapy (including a BTK inhibitor and a BCL-2 inhibitor) [52]. Despite a low CR rate, the majority of patients CLL was uMRD, and longer-term follow-up is needed to determine the durability of these responses and the effect of uMRD on partial responses. The concept of a uMRD PR was previously seen in patients treated with FCR, who often had residual splenomegaly, and these data demonstrate that traditional iwCLL response criteria do not account for these responses. Longer follow-up will be needed to ensure that these uMRD PR results translate to durable responses.

## 7. Ongoing Clinical Trials

There are many new clinical trials in development with MRD incorporated into their design. One notable trial is the MAJIC study, a phase III, prospective, multicenter RCT that will compare acalabrutinib and venetoclax vs. obinutuzumab and venetoclax in the first-line setting. Interestingly, in both arms, MRD will be used to guide the therapy duration, with a maximum therapy duration of two years. PFS will be the primary endpoint, and the rate of uMRD will be included as a secondary endpoint. The highly anticipated study will ultimately aim to provide additional information on the role of MRD in guiding therapy duration via NGS-based MRD assessment, and enrollment has been completed [53]. The phase 3 CLL17 trial will assess the use of ibrutinib, IV, or VO for frontline treatment of CLL, and U-MRD rates, as well as MRD levels, in the PB at different time points will be reported as secondary outcomes [54]. These pivotal trials will add to the growing literature demonstrating the importance of UMRD in patients treated with finite treatment regimens.

BTK degraders are another treatment modality under development. With a different mechanism of action to BTK inhibitors, they may have the ability to overcome the drug resistance seen in BTK inhibitors. Examples include BGB-16673, ABV 101 [55], and NX-5948, which are undergoing phase 1 and 2 clinical trials [56,57]. Currently, these drugs are being studied for use in continuous monotherapy, and further information regarding MRD use will be needed. Additionally, epcoritamab is an anti-CD3-CD20 bispecific antibody that recruits T-cell effector functions and is being studied in ongoing phase 1 and 2 clinical trials. The study is employing MRD4 in the BM in the absence of progression at 12 weeks as a primary outcome [58].

## 8. MRD: What Are the Next Steps?

The data collected to date show that achieving uMRD at the end of treatment, as defined by the iwCLL guidelines, can provide important prognostic information for chemoimmunotherapy- or venetoclax-based combination regimens in both frontline treatment and relapsed/refractory CLL treatment. UMRD, in these settings, serves as an independent predictor of improved survival across most studies. Currently, MRD assessment, if readily available, may be useful in clinical practice to guide expectations regarding PFS duration for fixed-duration venetoclax-based regimens, although it is not routinely recommended. In contrast, outcomes of continuous treatment with BTK inhibitors are excellent, despite low rates of uMRD, and therefore, routine MRD testing is not recommended in this setting.

As it relates to IV combination therapy, the results of using MRD-guided IV in the FLAIR trial are certainly promising. With this approach, it is notable that for patients with high-risk, IGHV-unmutated disease, there were substantial improvements in PFS and OS, though this was compared to FCR, which is not the standard of care for patients with uIGHV in the United States. Similarly, in CAPTIVATE, comparisons could not be made between the two cohorts to determine whether MRD-guided therapy improves upon outcomes compared to fixed-duration treatment. In CAPTIVATE, the MRD-guided cohort consisted largely of patients with high-risk features, including del17p, del11q, complex karyotype, TP53 mutation, and unmutated IGHV. Despite this, the rates ofuUMRD and PFS remained high, which reflects an area of future exploration of combination therapies for these patients. This contrasts with the outcomes in AMPLIFY, which excluded patients with del17p and TP53 mutations, where, numerically, the rates of uMRD were much lower. Longer follow-up from AMPLIFY will help to understand the role of EOT UMRD with this regimen. Data from the upcoming MAJIC trial will also add to our understanding of the role of MRD-guided therapy as a treatment option. Additionally, the addition of obinutuzumab to a BCL2i/BTKi combination has demonstrated increased depth of response, including UMRD responses, across several trials, but the use of triplets has been hampered by an increased risk of toxicity, particularly infections [33,42,59]. In this setting, how do we manage the risk of toxicity with the potential goal of deepening responses? The upcoming CLL16 trial will help to answer this question with randomized clinical trial data [42].

Understanding the rate, duration, and depth of MRD can provide insight into the optimal use of MRD to guide treatment. MRD kinetics are highly specific to both the treatment regimen and the presence or absence of high-risk genomic features (unmutated IGHV and del17p/TP53 mutations). For instance, in the VR arm of the MURANO trial, while high-risk patients were able to achieve high rates of uMRD in the relapsed/refractory setting, there was a faster doubling time after treatment completion and decreased PFS compared to patients without high-risk features, demonstrating that MRD kinetics may provide insight into the durability of response in fixed-duration regimens. This is also supported by ΔMRD400 in BOVEN, though more information regarding this measure and the associated genetic features are unknown. In GLOW, patients with unmutated IGHV receiving IV who achieved uMRD had a higher PFS rate than those who did not achieve uMRD.

Randomized trials demonstrating that MRD-guided treatment improves outcomes over fixed-duration or continuous treatment of patients with CLL are yet to be completed. Furthermore, if MRD-guided treatment is pursued, there is still a gap in knowledge regarding whether to re-initiate treatment based on dMRD alone. The duration of treatment remains an ongoing question, and for venetoclax-based therapies, 1–2 years appears to be appropriate for most patients to maximize response while maintain a time-limited treatment duration. MRD-guided strategies have the potential to maximize responses for most patients to maximize responses while minimizing drug exposure and possible resistance and toxicity. While the tailoring the duration of therapy, like in the FLAIR trial, is attractive, there are still patients whose disease may not reach the status of uMRD, and continuous therapy may be considered in these scenarios if this treatment strategy is shown to be effective. Finally, there may need to be different strategies for MRD-guided therapies based on genetics features, as we have seen lower rates of uMRD responses in patients with mIGHV, whose outcomes continue to be excellent, despite these lower UMRD rates across trials.

The clinical relevance of MRD is also variable by treatment, as highlighted by the difference in prognostic utility between continuous regimens and venetoclax-based combination regimens. While trials conducted thus far suggest that uMRD kinetics may be important with regard to high-risk patients, more research is needed on specific treatment regimens. For patients with high-risk disease, it is also important to highlight the potential of triplets vs. doublets. In the phase 2 AVO trial, high rates of uMRD were seen in patients with del17p/TP53 mutations [40]. Similar findings were seen in the subgroup enrolled in BOVEN [41]. Currently, there is debate regarding whether fixed-duration therapy is appropriate for patients with high-risk disease, and these data will demonstrate whether these responses are durable, and potentially what retreatment strategies are feasible.

The utility of uMRD as a surrogate endpoint is attractive, given the long overall survivals for patients with CLL. However, the use of MRD for routine clinical practice remains complicated and dependent on its intended use, the therapeutic regimen, and the treatment setting, and is not “one size fits all”, as is the case in other disease states, such as acute lymphoblastic leukemia and chronic myelogenous leukemia. Additionally, the interpretation of MRD results across clinical trials is challenging, given the differences in timing, the method of testing, and the definition of uMRD. In the era of targeted therapies, there are limited data demonstrating an overall survival benefit (though further follow-up is needed), so understanding its impact on long-term survival is limited.

One barrier to incorporation into clinical practice involves the detection method of MRD itself. Trials involving MRD vary widely in terms of the strategy of testing and often involve the testing of both the peripheral blood and the bone marrow, which may not be practical outside of a study setting. The cost, availability, and standardization of MRD detection techniques need to be optimized prior to their incorporation into routine care. Additionally, it is important to define how we are using MRD testing to inform clinical care: should we be basing the duration of treatment on MRD, as conducted in BOVEN and FLAIR? What do we do with a positive result after 12 months of venetoclax-obinutuzumab therapy? These and other important questions need to be answered before we can expect MRD to be utilized in routine clinical practice.

Many questions need to be answered prior to use of this approach, such as how and when MRD testing should be performed, as well as for which patients. For instance, do patients with high-risk genomic features benefit more from an MRD-guided treatment duration? What rate of UMRD is optimal for making these decisions? Is it best for use in the relapsed/refractory or frontline setting, or both? How do patients view UMRD testing, and does this add to treatment-related anxiety? Many ongoing clinical trials will provide additional information to answer these questions. As it stands, NCCN guidelines do not yet recommend the use of MRD for treatment decisions in the management of CLL [4].

## 9. Conclusions

In summary, MRD has been established as a valuable prognostic tool in CIT- and venetoclax-based combination regimens. Its use in these clinical settings would not be unreasonable, although it is not yet routinely recommended in clinical practice. Given that MRD-based decisions for clinical practice are likely to be complex and patient-specific, MRD assessment should continue to be incorporated into clinical trials to help answer the questions of how, for whom, and when MRD assessment is best utilized.

## Figures and Tables

**Table 1:** Comparison of the MRD testing modalities currently employed.

Modality	Methods	Limit of Detection (LOD)	Advantages	Disadvantages	Additional Information
FC	Cell sample treated with fluorescent antibodies Laser beam passed through sample Surface antigen detection via fluorescence pattern	10^−4^–10^−6^	- High throughput - Ability to analyze multiple cell markers simultaneously - Relatively short turnaround time	- Requires large, fresh samples - Highly skilled personnel needed	- Common assays comprise six markers: CD19, CD20, CD5, CD43, CD79b, and CD8 - Newer multi-colored assay techniques increase LOD
ASO-RQ-PCR	Fluorescently labeled DNA probe designed to detect gene of interest Several rounds of RT-PCR to amplify gene of interest Target DNA detection via fluorescence in droplets	10^−5^	- Real-time detection - Less time-consuming than other PCR methods - High sensitivity and broad range of detection	- Requires patient-specific primers - Temperature sensitivity (requires accurate melting temperature) - Expensive	- Identifies leukemia-specific rearrangements in IgH and T-cell receptor genes
NGS	Nucleic acids are extracted DNA sample converted to library of fragments Adapters are added to fragments Fragments are sequenced into genome Data analyzed and compared to reference genome	10^−6^	- High throughput - Ability to analyze large number of targets	- Requires pretreatment sample - Requires large data storage technologies	- ClonoSEQ assay specifically identifies rearranged IgH, IgK, and translocated BCL1/IgH and BCL2/IgH (J) sequences
dd-PCR	Sample partitioned into droplets using water–oil emulsion Droplets undergo PCR amplification Target DNA detection via fluorescence in droplets	10^−5^	- Quantitative measurement of target DNA without need for reference sample - Accurate and precise, especially when target concentration is low	- Requires patient-specific primers - Narrower dynamic range with larger sample sizes	- Useful in detection of ct - DNA for MRD quantification

**Abbreviations:** MRD: minimal residual disease; FC: flow cytometry; ASO-RQ-PCR: allele-specific oligonucleotide real-time quantitative polymerase chain; RT-PCR: reverse-transcription polymerase chain reaction; NGS: next-generation sequencing; dd-PCR: digital droplet polymerase chain reaction; ct-DNA: circulating tumor DNA.

**Table 2:** Summary of pivotal clinical trials utilizing MRD in patients with treatment-naive and relapsed/refractory CLL.

Treatment Setting	Trial	Phase	Primary Outcome	MRD Endpoint	MRD Detection Method	Treatment Duration	Treatment	Study Size	PFS	OS
**Previously untreated CLL**	*CLL14*	3	PFS	Secondary	ASO-RQ-PCR, NGS, FC	Time-limited, 12 cycles	VO CO	216 216	6 yr: 53% 6 yr: 22%	6 yr: 79% 6 yr: 69%
	*FLAIR*	3	PFS	Secondary	FC	Time-limited by uMRD stopping algorithm	IV FCR	260 263	3 yr: 97% 3 yr: 77%	3 yr: 98% 3 yr: 93%
	*CLL13*	3	uMRD, PFS	Primary	FC	Time-limited, 12 cycles with uMRD stopping algorithm in VOI cohort	FCR/BR VR VO VOI	229 209 229 231	4 yr: 62% 4 yr: 70% 4 yr: 82% 4 yr: 86%	4 yr: 94% 4 yr: 96% 4 yr: 95% 4 yr: 95%
	*GLOW*	3	PFS	Secondary	NGS	Time-limited, 12 cycles	IV CO	106 105	4.5 yr: 66% 4.5 yr: 25%	4.5 yr: 88% 4.5 yr: 78%
	*CAPTIVATE-FD*	2	CRR	Secondary	FC	Time-limited, 15 total cycles	IV	164	5 yr: 67%	5 yr: 96%
	*CAPTIVATE-MRD*	2	CRR	Secondary	FC	Continuous until MRD or disease progression	IV IV + I	31 32	4 yr: 88% 4 yr: 95%	4 yr: 100% 4 yr: 98%
	*AVO*	2	uMRD	Primary	FC, NGS	Time-limited, uMRD stopping algorithm	AVO	68	3 yr: 93%	NR
	*BOVen*	2	PFS	Primary	FC	Time-limited, 2 years	BOVen	39	2.5 yr: 97%	NR
	*E1912*	3	PFS	Exploratory	FC, ASO-RQ-PCR	Continuous	IR FCR	354 175	5 yr: 78% 5 yr: 51%	5 yr: 95% 5 yr: 85%
	*ELEVATE TN*	3	PFS	Exploratory	FC	Continuous	A AO CO	179 179 177	6 yr: 62% 6 yr: 78% 6 yr: 17%	6 yr: 76% 6 yr: 84% 6 yr: 75%
	*AMPLIFY*	3	PFS	Secondary	FC NGS	Time-limited	AV AVO FCR/BR	291 286 290	3 yr: 76.5% 3 yr: 83.1% 3 yr: 66.5%	3 yr: 94.1% 3 yr: 87.7% 3 yr: 85.9%
**Relapsed, refractory CLL**	*MURANO*	3	PFS	Secondary	ASO-RQ-PCR, FC	Time-limited, 2 years	VR BR	194 195	mPFS: 54 mo mPFS: 17 mo	5 yr: 82% 5 yr: 62%
	*CLARITY*	2	uMRD	Primary	FC	Time-limited, uMRD stopping algorithm	IV	45	NR	NR
	*VENICE*	3	CRR	Secondary	NGS	Time-limited, 2 years	V	258	mPFS: 28 mo	5 yr: 71%
	*CLL3X*	2	Safety	Exploratory	ASO-RQ-PCR	One-time	Allo-HSCT	90	5 yr: 38%	5 yr: 58%
	*TRANSCEND CLL004*	1–2	Safety	Exploratory	NGS, FC	One-time	Liso-cel	25	mPFS: 18 mo	NR

**Abbreviations:** MRD: minimal residual disease; UMRD: undetectable minimal residual disease; PFS: progression-free survival; mPFS: median progression-free survival; CRR: complete remission rate; FC: flow cytometry; ASO-RQ-PCR: allele-specific oligonucleotide real-time quantitative polymerase chain; NGS: next-generation sequencing; VO: venetoclax-obinutuzumab; CO: chlorambucil-obinutuzumab; IV: ibrutinib-venetoclax; FCR: fludarabine-cyclophosphamide-rituximab; BR: bendamustine-rituximab; VR: venteoclax-rituximaub; VOI: venteoclax-obinutuzumab-ibrutinib; AVO: acalabrutinib-venetoclax-obinutuzumab; BOVen: zanubrutinib-obinutuzumab-venetoclax; A: acalabrutinib; AO: acalabrutinib-obinutuzumab; VR: venetoclax-rituxumab; V: venetoclax; Allo-HSCT: allogeneic hematopoietic stem cell transplantation; Liso-cel: lisocabtagene maraleucel; NR: not reported.
